# Paramagnetism in *Bacillus* spores: Opportunities for novel biotechnological applications

**DOI:** 10.1002/bit.26501

**Published:** 2017-12-15

**Authors:** Ke Xu Zhou, Adrian Ionescu, Eamon Wan, Yeuk N. Ho, Crispin H.W. Barnes, Graham Christie, D. Ian Wilson

**Affiliations:** ^1^ Department of Chemical Engineering and Biotechnology University of Cambridge Cambridge United Kingdom; ^2^ Department of Physics University of Cambridge Cambridge United Kingdom

**Keywords:** *Bacillus*, paramagnetism, spores

## Abstract

Spores of *Bacillus megaterium*, *Bacillus cereus*, and *Bacillus subtilis* were found to exhibit intrinsic paramagnetic properties as a result of the accumulation of manganese ions. All three *Bacillus* species displayed strong yet distinctive magnetic properties arising from differences in manganese quantity and valency. Manganese ions were found to accumulate both within the spore core as well as being associated with the surface of the spore. *Bacillus megaterium* spores accumulated up to 1 wt.% manganese (II) within, with a further 0.6 wt.% adsorbed onto the surface. At room temperature, *Bacillus* spores possess average magnetic susceptibilities in the range of 10^−6^ to 10^−5^. Three spore‐related biotechnological applications—magnetic sensing, magnetic separation and metal ion adsorption—were assessed subsequently, with the latter two considered as having the most potential for development.

## 1 INTRODUCTION

*Bacillus* cells initiate sporulation as a protective mechanism in response to nutrient starvation. Successful formation of heat resistant, metabolically dormant spores typically requires mineral rich growth medium, including a requirement for manganese ions (Greene & Slepecky, 1972) (Charney, Fisher, & Hegarty, 1951). The importance of manganese for enzymatic activity in biological systems is well‐established (Culotta, 2000); however, while most microorganisms only require trace amounts of manganese, spores are unusual in that they accumulate a much greater amount than vegetative cells (Curran, Brunstetter, & Myers, 1943). Warth, Ohye, and Murrell (1963) reported that *Bacillus* spores could contain as much as 1.5 wt.% manganese. Such high levels of uptake suggest that manganese has an important function within spores. Many authors have explored the role of manganese ions in several of the spore's resistance properties (Ghosh et al., 2011; Levinson & Hyatt, 1964), as well as in germination (Levinson & Sevag, 1954; Levinson, Sloan, & Hyatt, 1958). However, only one group has reported the intrinsic magnetic properties conferred by the high manganese content in spores (Melnik et al., 2007).

Manganese is a chemically versatile transition metal, which can exist in several oxidation states ranging from +1 to +7. This allows manganese to display a wide variety of magnetic behaviors, from ferromagnetic manganese oxides (Du, Zhang, Sun, & Yan, 2009) to diamagnetic permanganate compounds (Goldenberg, 1940). The exact chemical state of manganese in the spore is not known, however, evidence suggests that Mn^2+^ ions compete with Ca^2+^ ions to form a chelate with dipicolinic acid (DPA) inside the spore core (Bailey, Karp, & Sacks, 1965). In this case, bacterial spores are very likely to be paramagnetic at room temperature, exhibiting a small attractive force toward permanent magnets.

The magnetic properties of spores may be of biotechnological value, particularly in sensing, and separation related applications. Current magnetic methods for the separation of spores require them to be tagged with antibody functionalized magnetic beads (Shields et al., 2012). However, with the exception of magnetotactic bacteria (Blakemore, 1975), most microorganisms tend to be diamagnetic, thus the intrinsic paramagnetism of spores may be sufficient to achieve efficient separation.

In sensory applications, most methods rely on a similar antibody functionalisation and tagging procedure (Gómez de la Torre et al., 2012; Wang et al., 2015) without exploiting the spore's intrinsic paramagnetic properties. Conceivably, potential cross‐species variance in the latter may be detectable by a sensitive magnetometer and used to identify dangerous species such as *B. anthracis*. However, increased understanding of the magnetic properties of spores is required before any applications can be considered. Accordingly, the aim of this work was to fully characterize the magnetic properties of three species of *Bacillus* spores, determining the type of magnetism that they exhibit, and determining the role of manganese in conferring this magnetism. Possible alterations to the magnetic properties as well as novel biotechnological applications are also explored.

## 2 Materials and Methods

### 2.1 Bacterial strains and sporulation conditions

Three lab strains of *Bacillus* were studied in the current work: *B. megaterium* QM B1551, provided by Prof. P. S. Vary (Illinois); *B. cereus* 569, provided by Prof. A. Moir (Sheffield, UK), and *B. subtilis* PS832, provided by Prof. P. Setlow (CT). The sporulation conditions and spore purification procedures followed were those described previously by Nicholson and Setlow (1990), Clements and Moir (1998) and Christie, Ustok, Lu, Packman, and Lowe (2010) for the sporulation of *B. subtilis*, *B. cereus*, and *B. megaterium*, respectively. The latter two species were additionally cultured in media containing manganese concentrations varying between 20 and 1,000 µM. In addition, the uptake of iron and cobalt ions in *B. megaterium* spores was investigated by culturing spores in media containing 100 µM of FeSO_4_ and CoCl_2_, respectively.

### 2.2 Sample preparation for magnetometer measurements

Clean spore suspensions, containing >95% spores (checked by optical microscopy), were concentrated to a paste‐like density by centrifugation. Between 100 and 150 µl of this paste was pipetted into the cap and body of a size 0 gelatine capsule, pre‐frozen to −80°C. This achieves a quick freeze and prevents the gelatine from extracting water from the spore paste. The frozen spore capsule was then lyophilized at −20°C and 250 mTorr pressure for 8 hr. Capsules were sealed when dry and weighed to determine the dry mass of spores within. To minimize any background signal, spore capsules were suspended within a straw supplied by Quantum Design (San Diego, CA) with the aid of two low paramagnetic glass rods. The straw was then sealed with cotton at both ends and Kapton™ tape at the lower end before being placed into the magnetometer for measurement.

### 2.3 Magnetic property measurements

The magnetic response of spore samples was measured in a superconducting quantum interference device (SQUID) (Quantum Design MPMS 2XL) for both field and temperature dependence. For field dependence, the temperature was set to 5 K and a full hysteresis sweep between 5 T and −5 T was conducted. In temperature dependence, zero field cooling measurements were conducted: the sample was first cooled to 2 K before a 0.1 T magnetic field was applied. The temperature was then increased to 300 K in 2 K intervals.

### 2.4 Imaging and spectroscopy

*B. megaterium s*pore samples were prepared for scanning electron microscopy (SEM) imaging and energy dispersive X‐ray spectroscopy (EDS). Around 1 ml of a high density (OD_600_ > 10) spore suspension was deposited on a Leit tab pre‐coated with poly‐L‐lysine to increase spore adhesion. After 5 min, the tabs were gently rinsed with distilled water to remove any unwanted chemicals and subjected to a fast freeze in liquid ethane to preserve the spore's morphological features. Frozen samples were lyophilized overnight at a fixed pressure of 10^−3^ mbar and a temperature programme of initially −90°C for 2 hr then subsequently increasing by 10° per hour up to a final temperature of 30°C. Lyophilized samples were then sputter coated with a thin layer (25–50 nm) of conductive carbon and stored in a dry location, at room temperature, prior to imaging. SEM imaging and EDS were undertaken using a FEI XHR Verios 460 (Hillsboro, OR) mounted with an AMETEK EDAX detector device (Mahwah, NJ).

### 2.5 Inductively coupled plasma–optical emission spectroscopy (ICP‐OES)

The lyophilized spore capsule samples prepared for magnetic measurements were submitted for ICP‐OES analysis to determine the manganese content in spores (Medac Ltd., Chobham, UK).

### 2.6 Spore demineralisation

Spores were demineralized by acid titration as previously described by Marquis and Bender (1985). A solution of 5 wt.% spores was titrated with hydrochloric acid over approximately 4 hr until the pH stabilized at 4.0. To ensure that all minerals were removed, the suspension was washed three times by centrifugation and resuspension in deionized water and the titration process repeated. All acid treated spores were prepared from the same culture batch as their non‐treated counterparts and were thoroughly washed in deionized water before conducting experiments.

### 2.7 Surface adsorption experiments

Approximately 0.2 g of MnCl_2_ or FeSO_4_ salt crystals were added to a 25 ml suspension of 1 wt.% *B. megaterium* spores and the solution was mixed well. Once the crystals had dissolved, 1 ml of 1 M sodium hydroxide solution was pipetted into the suspension, forming a colored precipitate. The suspension was then vortexed until no further oxidation occurred and the color of the precipitate was stable. A total of 2 ml of 2 M HCl was subsequently added to the suspension, which was incubated on ice overnight. Spores were separated from inorganic debris by repeated rounds of centrifugation and resuspension in deionized water. Suspensions were cleaned to give over 95% phase‐bright spores before experiments were conducted.

### 2.8 Theory

Paramagnetic materials possess at least one unpaired electron that can align with the externally applied magnetic field; however, the extent to which these electrons can align with the magnetic field is low as thermal excitation randomizes the spin orientation for most electrons and only a small proportion will align with the applied field. In most cases, the mass magnetisation, *M*, of a paramagnetic material depends linearly on the applied field, ***H***, according to,

(1) $$M=\chi_{m}H$$

where χ_m_ is the mass magnetic susceptibility (m^3^ kg^−1^). According to Shevkoplyas, Siegel, Westervelt, Prentiss, and Whitesides (2007), the magnetic susceptibility of the material, following conversion from centimetre‐gram‐second units to SI units, may be expressed as,

(2) $$\chi_{spore}=\frac{4\pi }{10^{3}}\rho_{spore}\frac{\Delta M}{\Delta H}$$

where χ_spore_ is the dimensionless magnetic susceptibility of the spore and *ρ*_spore_ is its density in kg m^−3^. At low enough temperatures or high enough fields, however, saturation occurs and the field dependence can be expressed using the Langevin function,

(3) $$M=Nm\mu_{B}[\coth(\frac{\mu_{B}{m\;\mu }_{0}H}{kT})-\frac{kT}{\mu_{B}{m\mu }_{0}H}]$$

where ***M*** is the mass magnetisation of the sample (A m^2 ^kg^−1^), *N* is the number of magnetic entities per unit kg of spores, *m* is the magnetic moment of each entity in Bohr magnetons, μ_B_ is the Bohr magneton constant (9.274 × 10^−24^ J T^−1^), *k* is the Boltzmann constant (1.381 × 10^−23^ m^2^ kg s^−2^ K^−1^), µ_0_ is the magnetic permeability of free space (4π × 10^−7^ H m^−1^), and **H** is the applied magnetic field (A m^−1^). The magnetic moment, *m*, should indicate the oxidation state of the manganese contained within the spores and the number of magnetic entities, *N*, can be converted into a weight percentage of manganese, *w*_Mn_, according to,

(4) $$W_{Mn}=\frac{N}{6.02\times 10^{26}}\times 54.938\times 100\%$$

The magnetic susceptibility of paramagnetic materials is found to obey Curie's law,

(5) $$\chi_{m}=\frac{C}{T}$$

where *C* is the Curie constant given by Cullity (1972) as,

(6) $$C=\frac{\mu_{0}N{\left(m\mu_{B}\right)}^{2}}{3k}$$

Siegel et al. (2006) developed a microfluidic device (Supplementary Figure S1) that was used to separate superparamagnetic beads using a weak electromagnetic field. The theoretical equations later derived by Shevkoplyas et al. (2007) were employed to assess the feasibility of exploiting the magnetic properties of spores for separation. The setup consists of a 40 µm wide channel sandwiched between two electromagnets. The distance between the surface of the channel and the center of the electromagnet is 70 µm. In their analysis, the electromagnet was modeled as a single electrical wire carrying current *I*. According to Shevkoplyas et al. (2007), the displacement time, *t*, required for a spore of radius *R* to move through a liquid of viscosity µ from its initial position, *x*_i_, to the final position, *x*_f_, is given by

(7) $$t=-\frac{1}{4\beta }\left(x_{f}^{4}-x_{i}^{4}\right)$$

where

(8) $$\beta =-\frac{\chi_{spore}R^{2}\mu_{0}I^{2}}{18\pi^{2}\mu }$$

### 2.9 Statistics

Statistical analysis was performed using Microsoft Excel (Redmond, WA). The method of least squares was used to fit experimental data to the corresponding equations. At each temperature or magnetic field strength, the magnetisation value was evaluated as the average of three scans; the errors associated with each measurement were evaluated by the magnetometer's inbuilt software. These errors were then used to estimate the uncertainties associated with the least squares fitting and hence the error in the fitting parameters.

## 3 RESULTS

### 3.1 Magnetic behavior of *B. megaterium* spores

**Figure 1:**
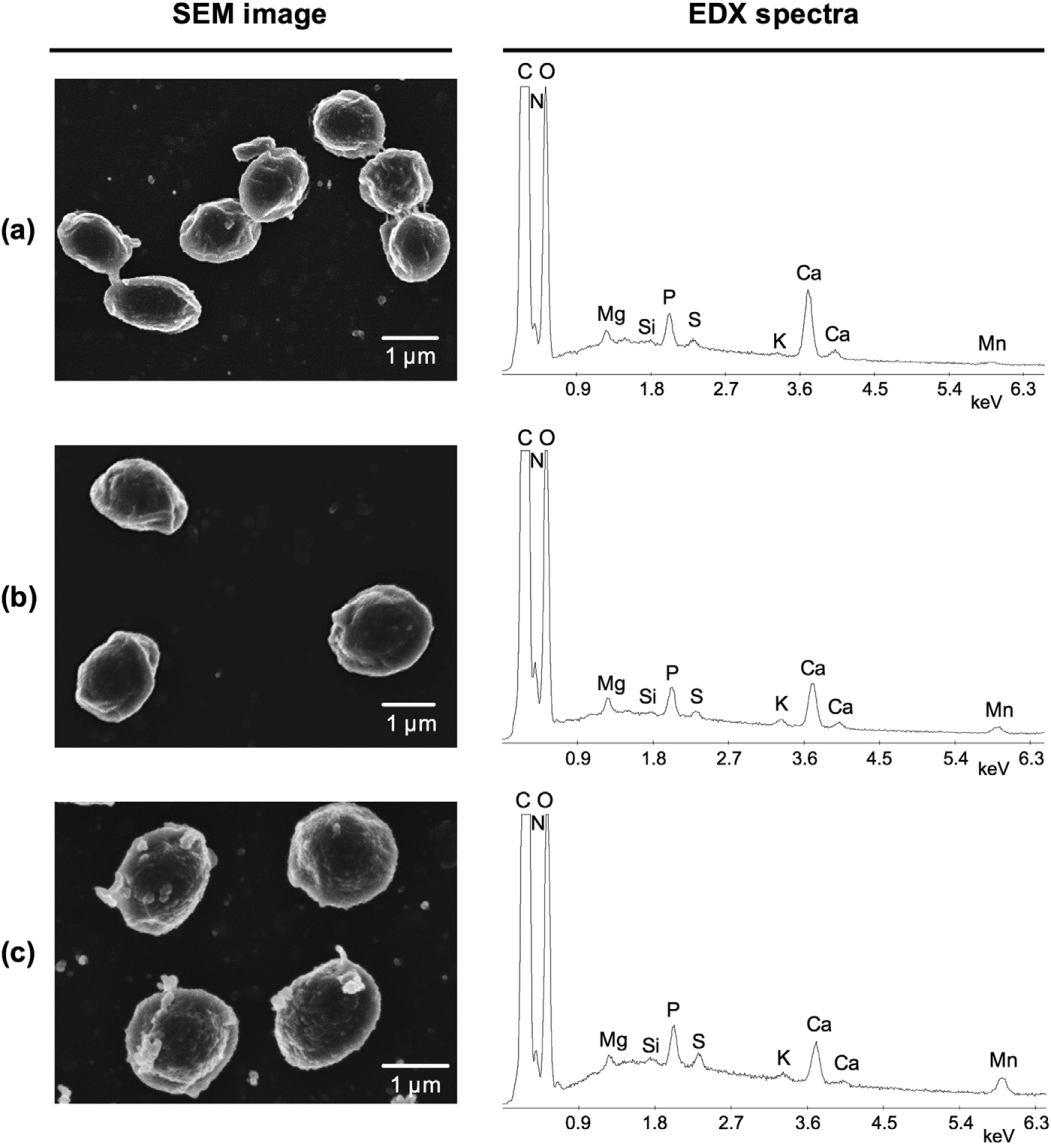
EDX spectra and corresponding SEM images for *B. megaterium* spores cultured in media with differing manganese concentrations: (a) 20 µM (standard); (b) 100 µM; and (c) 1,000 µM

It is well‐known that bacterial spores contain a wide range of inorganic elements when cultured in media with an adequate metal balance (Murrell, 1969). The EDX spectrum in Figure 1a shows that *B. megaterium* spores grown in standard supplemented nutrient broth (SNB) possess Ca, P, Mg, K, and Mn as their main inorganic constituents. This result is in agreement with those reported by Murrell (1967) with the exception that the spectrum contains no trace of Fe or Na. These elements are most likely present in quantities below the detection limit for the EDX detector (typically 0.1 wt.%). In *B. subtilis*, Granger, Gaidamakova, Matrosova, Daly, and Setlow (2011) used ICP‐OES to find the spore's iron abundance to be around 0.005 wt.%, two orders of magnitude lower than the typical amount of manganese present. Additionally, good agreement is found between the relative size of the EDX spectra peaks and the relative proportions of each element determined by Curran, Brunstetter, and Myers (1943) and Murrell (1967) for a variety of *Bacillus* species and strains.

The larger Mn peaks in Figures 1b and 1c indicate that the developing spores had taken up additional manganese when produced in higher manganese concentration media. Furthermore, as the Mn peak becomes larger the Ca peak decreases while all other peaks remain similar in size. The former observation is in agreement with the hypothesis that calcium and manganese ions compete for sites in the spore, as suggested previously by Slepecky and Foster (1959) and Levinson and Hyatt (1964). When spores were cultured in the presence of other divalent transition metal salts, no uptake was detected by EDX (data not shown). This result suggests that the uptake mechanism is specific to manganese and if spores do accumulate other transition metal ions, the amounts are below the detection limit for EDX analyses.

**Figure 2:**
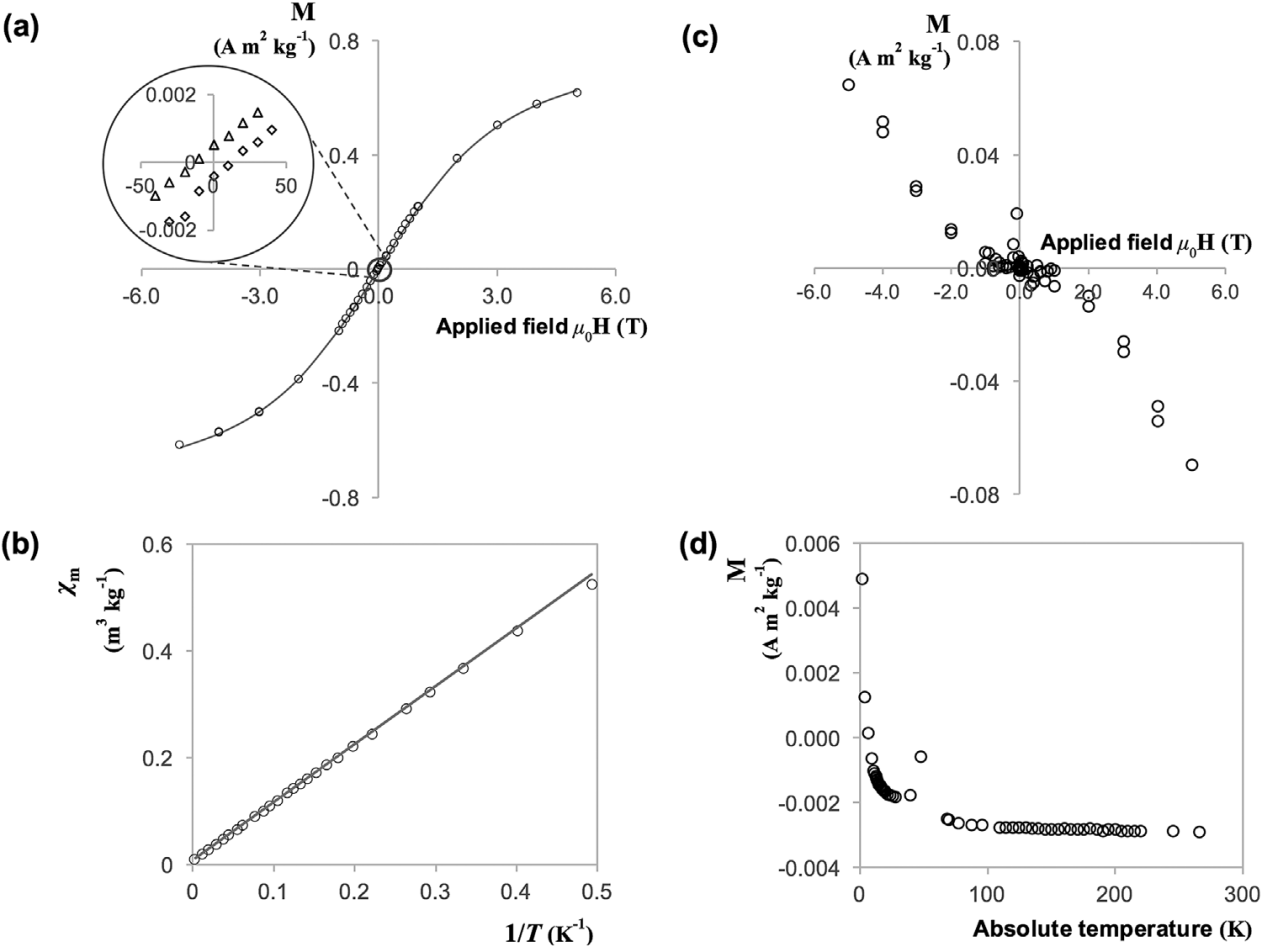
SQUID scans for *B. megaterium* QM B1551 spores, (a) and (b), and vegetative *B. cereus* cells, (c) and (d). (a) Field sweep measurement at 5 K, with the solid line corresponding to Equation (3) fitted with parameters *N *= 1.55 × 10^22^ ****kg^−1^ and *m *= 5.9; (b) Temperature sweep data at a magnetic field of 1,000 Oe; the solid line corresponds to equation (5) fitted with parameter *C *= 1.06m^3^ K kg^−1^; (c) Field dependency measurement at 5 K; (d) Temperature dependency measurement at a magnetic field strength of 1,000 Oe. The circled region in (a) is magnified to show that spores do not exhibit hysteresis; the apparent coercive field of 10 Oe arises due to trapped flux in the superconducting coils. Key: circles, data for *B. megaterium* spores; in the magnified region, triangles refer to virgin curve up and the hysteresis sweep up; diamonds refer to the hysteresis sweep down. Error bars indistinguishable when plotted

Superconducting quantum interference device (SQUID) measurements of *B. megaterium* spores yielded the magnetisation and temperature dependence data presented in Figures 2a and 2b, respectively. The shape of the field sweep curve in Figure 2a is characteristic of a strong paramagnetic material, reaching saturation at high fields with no signs of hysteresis. The apparent hysteresis observed with a small coercive field of 10 Oersted is an artefact that arises as a result of trapped flux in the superconducting coils of the magnetometer. Figure 2a shows that *B. megaterium* spores, cultured under standard conditions, possess 1.55 × 10^22^ manganese particles per kilogram of spores, corresponding to 0.14 dry wt.%. Furthermore, both Figures 2a and 2b confirm that the spores are paramagnetic over the temperature range from 2 to 300 K, with an effective magnetic moment of approximately 5.9 Bohr magnetons. This suggests that the ionic state of the manganese is 2+ (Figgis & Lewis, 1964) and is very likely to be part of a tetrahedral or octahedral complex.

### 3.2 Magnetic properties of spores of other species and vegetative cells

An observable paramagnetic response in the presence of a magnetic field gradient is not a property exclusive to *B. megaterium* spores. Melnik et al. (2007) first observed and demonstrated paramagnetic resembling effects in *B. atrophaeus*, *B. thuringiensis*, and *B. cereus* spores. In this work, *B. cereus* and *B. subtilis* spores were also found to be paramagnetic, however, the magnitude of magnetisation for the three *Bacillus* species were different. The differences in magnetisation were not only related to the quantity of manganese present in spores of each species, but unexpectedly, the magnetic moments were also different across species (Table 1). *Bacillus cereus* spores have a lower average magnetic moment of 5.1 Bohr magnetons, indicating that each magnetic entity possesses four unpaired electrons instead of five; meaning that, although manganese is still responsible for paramagnetism in *B. cereus* spores, its oxidation state is more likely to be 3+ rather than 2+. In contrast, the magnetic moment of *B. subtilis* spores, although lower than that of *B. megaterium*, still falls within the range of magnetic moments observed for Mn^2+^. These results indicate that manganese ions of various valencies are responsible for the paramagnetic behavior observed in all three *Bacillus* species.

**Table 1:** Best‐fit parameters for field and temperature sweep measurements showing the manganese content (*N*) and magnetic moment (*m*) in spores of three species of *Bacillus*

	Field sweep (*T* = 5 K)		Temperature sweep (*H* = 1,000 Oe)	
Species	*N* (×10^22 ^kg^−1^)	*m* (µ_B_)	*C* (×10^−6^ m^3^ K kg^−1^)	*m* (µ_B_)
*B. megaterium*	1.55 ± 0.08	5.9 ± 0.2	1.06 ± 0.03	5.7 ± 0.3
*B. cereus*	2.3 ± 0.1	5.1 ± 0.2	1.68 ± 0.02	5.3 ± 0.2
*B. subtilis*	4.2 ± 0.3	5.5 ± 0.3	3.22 ± 0.07	5.7 ± 0.2

Field sweep data have been fitted to Equation (3). Temperature sweep data have been fitted to Equation (5) and the corresponding *m* was calculated using Equation (6).

The magnetic response of vegetative *Bacillus* bacterial cells was also measured with the SQUID magnetometer. Figures 2c and 2d show the typical shape of field and temperature dependency measurements obtained. Evidently, vegetative cells respond very differently to magnetic fields. As expected, the negative slope of field dependency measurements indicates that vegetative cells are diamagnetic at 5 K, with temperature dependency measurements confirming this attribute over a wider range of temperatures. Figure 2d also shows a characteristic paramagnetic tail at low temperatures. However, the strength of a diamagnetic signal is only affected by the strength of the applied field and does not exhibit temperature dependency. The mass of vegetative cell samples was typically 10 mg—approximately 25% of the weight of the capsule in which it was contained. Since the gelatine capsule is weakly paramagnetic, it is likely that at lower temperatures its signal dominated that of the diamagnetic cells resulting in the paramagnetic tail observed in Figure 2d.

### 3.3 Effect of culture medium inorganic salt concentration on spore paramagnetic properties

**Figure 3:**
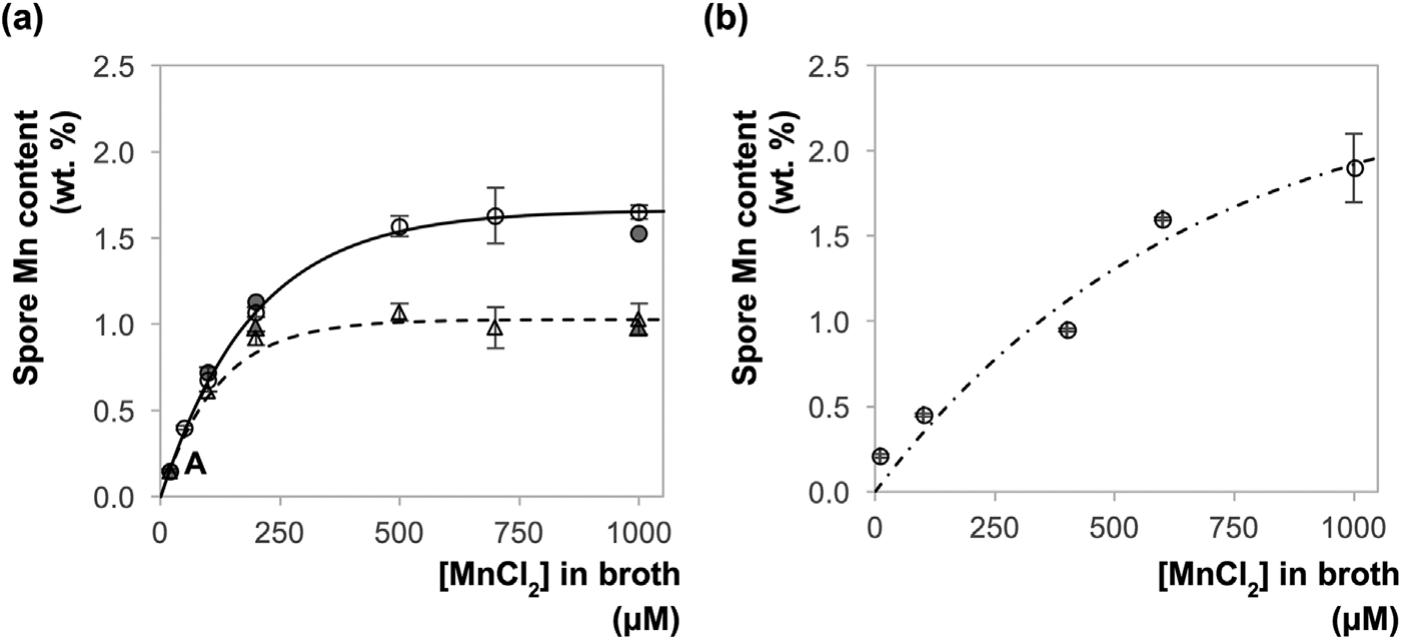
Plot showing the accumulation of manganese with increasing concentration of MnCl_2_ in the culture medium of (a) *B. megaterium* spores; (b) *B. cereus* spores. Key: circles, untreated spores; triangles, acid treated spores; open symbols, data obtained by SQUID measurements; solid symbols, ICP‐OES measurement for the closest corresponding solid symbol sample. The position denoted ‘A’ contains three data points that overlap. Lines show the fit of data sets to Equat**i**on (9): solid locus, untreated *B. megaterium* spores, *a*_1_ = 1.66 wt.% and *a*_2_ = 196 µM; dashed locus, acid‐treated *B. megaterium* spores, *a*_1_ = 1.03 wt. % and *a*_2_ = 119 µM; dot‐dashed locus, untreated *B. cereus* spores, *a*_1_ = 2.46 wt.% and *a*_2_ = 661 µM. Tables containing presented data are provided in the supplementary information (Supplementary Tables S1–S3). The error bars indicate the uncertainty derived from SQUID measurements

The concentration of manganese in culture media had a strong impact on the strength of the measured signal as it determines the amount of manganese that the mature spore contains (Supplementary Tables S1 and S2). As the manganese content increased, the corresponding magnetic moments decreased from 5.9 to 5.1 and from 5.1 to 3.8 for *B. megaterium* and *B. cereus* spores, respectively. According to Figgis and Lewis (1964), lower magnetic moments indicate that the predominant manganese valency in spores with enhanced manganese content is increased by one. As the drop in magnetic moment occurs gradually with manganese concentration, it indicates that more than one oxidation state of manganese is present in the spore and that the relative proportions of each is determined by the concentration of manganese during spore formation. Given the range of magnetic moments observed, it is likely that *B. megaterium* spores contain a combination of the 2+ and 3+ oxidation states and *B. cereus* spores a combination of 3+ and 4+, however, the presence of smaller quantities of other oxidation states cannot be ruled out. Figure 3 illustrates the effect of culture medium manganese concentration on the manganese content of *B. megaterium* and *B. cereus* spores. The accumulation of manganese in both acid treated and untreated spores can be adequately described by an exponential decay function of the form

(9) $$W_{Mn}=a_{1}\left(1-e^{-[Mn]/a_{2}}\right)$$

where [Mn] is the concentration of manganese in the culture medium, and *a*_1_ and *a*_2_ are constants which represent the saturation manganese weight percent and the culture concentration which yields 63% of the saturation value, respectively. At low manganese concentrations (<100 µM), the quantity of manganese uptake is linear and almost identical for both acid treated and untreated spores. At higher concentrations, the manganese content tends asymptotically toward 1.0 and 1.6 wt.% for acid treated and untreated spores, respectively. The accuracy of values is confirmed by the agreement with ICP‐OES measurements for selected samples, as observed in Figure 3. Although the Langmuir isotherm is equally adequate in modelling the data in Figure 3, the isotherm describes the surface adsorption of materials rather than the combined effects of adsorption and absorption. Further analysis of the data using the Langmuir isotherm (Supplementary Figure S3) can be found in the discussion.

The consistently lower manganese content in acid‐treated spores indicates that manganese is externally bound and removed by this treatment. Furthermore, it suggests that spores accumulate manganese through two mechanisms, giving rise to the two asymptotes observed in Figure 3a. The lower asymptote is likely to be determined by the manganese transport process that is active during sporulation, whereas the higher asymptote is a physical limit imposed by adsorption on to the surface of the spore.

The manganese content in *B. cereus* spores did not reach saturation at 1,000 µM as observed with *B. megaterium*. Fitting the data to Equation (9) predicts a higher asymptotic value, of 2.46 wt.%, attained at concentrations exceeding 3,000 µM. This may be a result of the large exosporium present in *B. cereus* spores, which may provide a greater number of sites for the adsorption of manganese ions. The average exosporium surface area of *B. cereus* spores is approximately 25% larger than that of *B. megaterium* spores (Xu Zhou, Wisnivesky, Wilson, & Christie, 2017).

In contrast, measurements made on spores cultured in the presence of other divalent ions, that is, Fe^2+^ or Co^2+^, were not appreciably different to those grown under standard conditions (Supplementary Figure S2). This suggests that developing *B. megaterium* spores have some form of specificity toward the uptake of manganese ions and neither adsorb nor absorb significant amounts of iron or cobalt. A similar observation for the uptake of iron ions was reported by Granger et al. (2011) in *B. subtilis*, but this result for cobalt contradicts the findings of Slepecky and Foster (1959). In the latter, *B. megaterium* spores grown in media containing 680 µM Co^2+^ possessed 0.14 wt.% cobalt. This difference may be caused by the lower concentration of cobalt (150 µM) used in this work.

### 3.4 Magnetic susceptibility at room temperature

**Figure 4:**
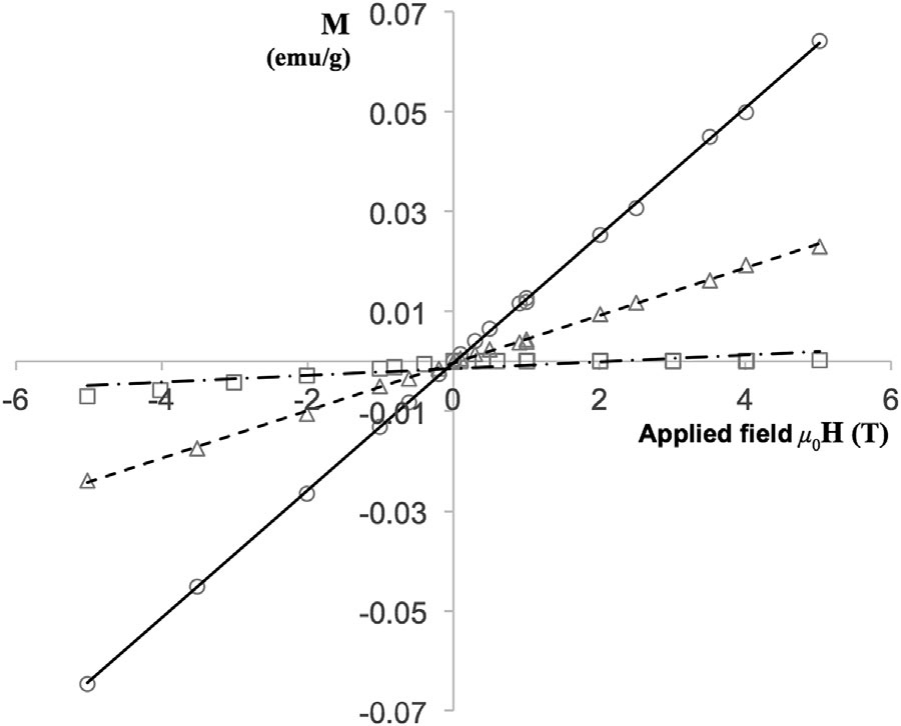
Magnetisation at 300 K of three *B. megaterium* spore samples cultured in different concentrations of MnCl_2_. Key: squares, 20 µM MnCl_2_; triangles, 50 µM MnCl_2_; circles, 100 µM MnCl_2_. Lines represent the line of best fit to Equation (2); the gradients are 20 µM, 0.68 × 10^−6^; 50 µM, 4.77 × 10^−6^; 100 µM, 1.28 × 10^−6^. Error bars indistinguishable when plotted

The spore's magnetic susceptibility at 300 K can be determined from the slope of the magnetisation chart. Figure 4 shows the magnetisation behavior of *B. megaterium* spores cultured in media with three different concentrations of MnCl_2_. All three data sets show linear behavior and the magnetic susceptibility can be calculated from Equation (2) (using SI units) as 1.0 × 10^−6^, 7.2 × 10^−6^, and 1.9 × 10^−5^ for 20, 50, and 100 µM MnCl_2_, respectively. As the magnetic moments do not reach saturation at 300 K, these susceptibility values are indicative of spores of the *Bacillus* genus; therefore, at room temperature, the average magnetic susceptibility of *Bacillus* spores lies in the range 10^−6^ and 10^−5^.

### 3.5 Surface adsorption

Figure 3 shows that the internal manganese content of *B. megaterium* spores saturates at approximately 1.0 wt.% and a further 0.6 wt.% could adsorb onto its surface. However, when acid treated spores with a saturated core were remineralized in a solution containing 500 and 700 µM MnCl_2_, the manganese content, measured by ICP‐OES, was higher than expected; at 1.74 and 2.17 wt.%, respectively. In this case, presumably more manganese could adsorb onto the spore's surface because, unlike in the culture media, there were no other competing ions.

**Figure 5:**
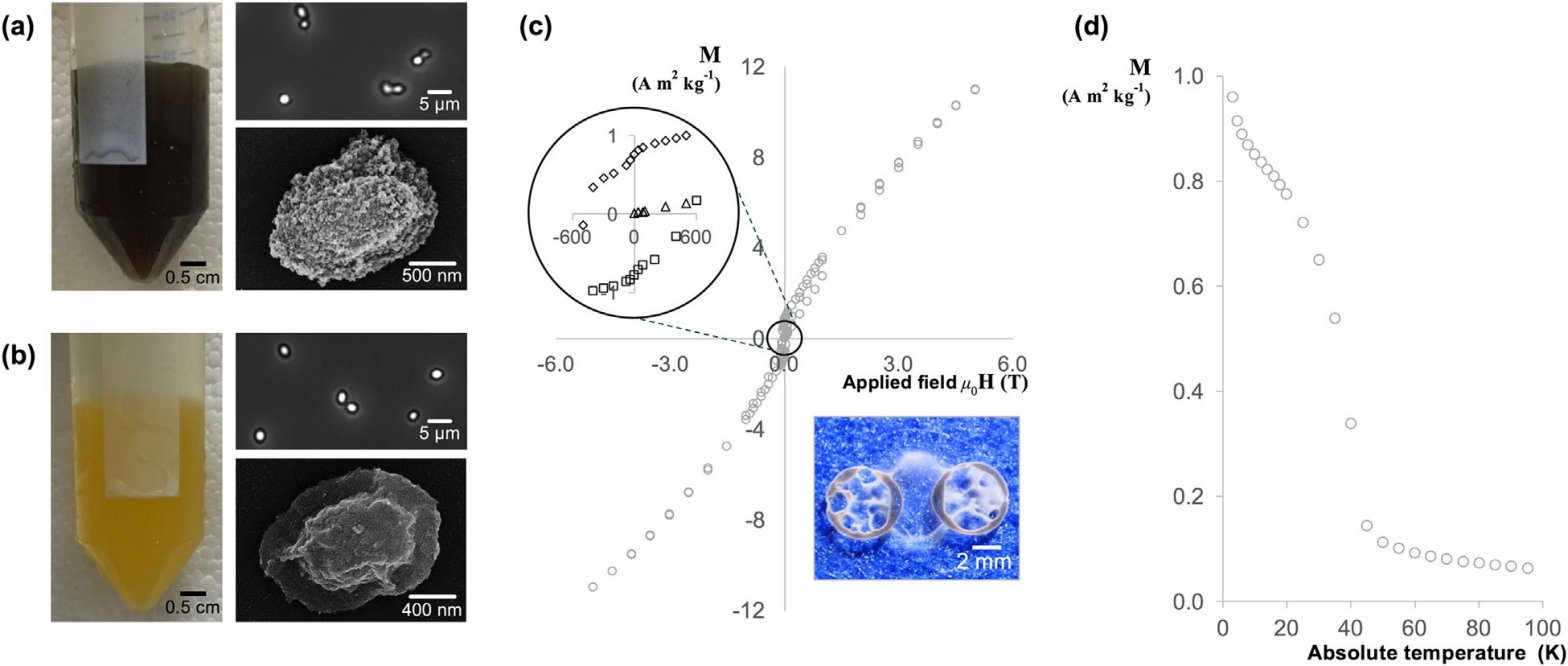
Magnetic measurements and images depicting the color and appearance of *B. megaterium* spores that have undergone the adsorption procedure described in section 2.4.3. (a) manganese coated spores; (b) iron coated spores; (c) field dependency measurement for manganese coated spores at 5 K; the image shows a sample of spores aligned along the magnetic field lines between two circular neodymium magnets; (d) temperature dependency measurement for manganese coated spores with a magnetic field strength of 1,000 Oe. In (a) and (b) the images show: left, color of the spore suspension; top right, phase contrast image of the spores; bottom right, SEM image of the spore. Error bars indistinguishable when plotted

The surface adsorption procedure resulted in a change to the spore's magnetic behavior. The visual appearance of *B. megaterium* spores that had undergone the adsorption procedure is shown in Figures 5a and 5b. Under phase contrast microscopy, the spores appear to be undamaged and phase bright, indicative of their dormancy; however, at higher magnification, scanning electron microscopy reveals that the surface has been coated with multiple layers of acid‐insoluble inorganic material, forming a shell surrounding the spore. This material is likely to be an oxide of the corresponding metal and the color emitted by the samples suggest that both manganese and iron are in the 3+ oxidation state.

The manganese coated spore sample possesses a strong magnetic signal at 5 K, displaying hysteresis and a very large coercive field of approximately 500 Oersted. To illustrate their magnetic strength, even at room temperature, Figure 5c contains an image of these spores aligning with the magnetic field lines between two circular neodymium magnets; a behavior that resembles that of iron filings. The shape of Figure 5c is uncommon as it derives from two contributions—a paramagnetic component from within the spore and a surface contribution that appears to be ferromagnetic. Figure 5d reveals that the surface manganese undergoes a transition at temperatures above 43 K, which corresponds to the Curie temperature of Mn_3_O_4_ (Boucher, Buhl, & Perrin, 1971). These results strongly indicate that the manganese shell surrounding the spore exhibits bulk ferromagnetic properties at 5 K; nevertheless, an antiferromagnetic contribution, due to the presence of uncompensated spins on the surface, cannot be entirely excluded (Cooper et al., 2013).

The iron coated spore sample demonstrates that the adsorbate is not limited to manganese. These spores bear the distinctive bright yellow color of a hydrated iron oxide‐hydroxide (FeO(OH) · nH_2_O). Figure 5b reveals that the crystals form thin rods and aggregate in a foliated manner, fitting the description for goethite (Cornell & Schwertmann, 2004a). In this case, the iron coated spores would be antiferromagnetic with a Néel temperature of 400 K (Cornell & Schwertmann, 2004b); however, further analyses are required to confirm the identity of the iron compound.

## 4 DISCUSSION

To date, there have only been two accounts reporting the intrinsic magnetic properties of bacterial spores (Melnik et al., 2007; Sun, Zborowski, & Chalmers, 2011). This work provides the first comprehensive characterisation of these properties in *Bacillus* spores and explores the extent to which they can be altered. In agreement with previous results (Warth et al., 1963), manganese is the only non‐diamagnetic element that accumulates to any significant amount in spores and SQUID measurements have confirmed that this manganese is paramagnetic at all temperatures up to 300 K. Similar to Sun et al. (2011), we determined that several oxidation states of manganese (2+, 3+, and 4+) are present in spores; however, our results indicate a lower manganese content, in the range of 0.2 wt.% rather than 10 wt.%. Increasing the concentration of manganese in the culture medium raises the manganese content in spores approximately 10‐fold. Nevertheless, the force experienced by these spores in a magnetic field would still be several orders of magnitude smaller than that experienced by ferromagnetic entities. With this in mind, potential applications associated with spore paramagnetism include:

(i) Sensing: Manganese was found to be the cause for paramagnetism in all three *Bacillus* species studied, yet their respective SQUID measurements presented some clear differences. The chemical state and environment in which the manganese ions exist in the spore differs across the species—under standard conditions, not only do the spores contain different amounts of manganese, their magnetic moments also range between 5.1 and 5.9 µ_B_. These differences may be useful for the detection of more harmful, pathogenic species such as *B. anthracis* or *Clostridium difficile*. There are, however, two major obstacles to overcome. The first concerns sensitivity; the device must be sensitive enough to detect the magnetic signal from, typically, a small quantity of spores. This obstacle is perhaps the easiest to overcome as there are commercial paramagnetic oxygen sensors that can measure the concentration of oxygen at room temperature. According to Wills and Hector (1924), the magnetic susceptibility of oxygen at room temperature is of the order of 10^−7^, suggesting that these devices would also be capable of detecting the magnetic signal emitted by spores. The second obstacle concerns differentiation; the ability to distinguish specific spore species or strains from a mixture of spores and impurities. Although most impurities will possess a very different magnetic susceptibility to spores, it will be challenging to differentiate between spore species. In particular, the spore's magnetisation does not saturate at room temperature and therefore it will not be possible to obtain magnetisation curves such as those found in Figure 2a. Furthermore, sporulation conditions have a strong influence on the spores’ magnetic properties, causing their magnetic susceptibility to vary over one order of magnitude. Given the difficulty of overcoming this challenge, it is unlikely that magnetic fields could be used to discern between different spore species.

(ii) Separation: A microfluidic device designed to expose spores in aqueous suspension to an open gradient permanent magnet has been demonstrated to capture and deposit *B. atrophaeus* spores onto a glass slide (Melnik et al., 2007). To assess further the theoretical considerations associated with spores’ intrinsic paramagnetism for separation applications, a microfluidic device such as that presented by Shevkoplyas et al.(2007) may be considered. To estimate the shortest time required for separation, the highest values for magnetic susceptibility (10^−5^) and electric current (1 A, maximum current used in their work) will be assumed. Furthermore, spores may be approximated as spheres with radius 0.5 µm. The liquid viscosity is expected to be around 10^−3^ Pa s. Using these values, Equation (7) estimates that, under the influence of a weak magnetic field, it would take over 166 hr for the spores to reach the sidewall from the midpoint of the microfluidic channel. At this timescale, separation would not be feasible, however it should be noted that the magnetic field generated in the channel is weak, at approximately 2.5 mT for 1 A current. For comparison, a typical permanent neodymium magnet has a remanence between 0.2 and 1.2 T. As the wire is only a proxy for the electromagnet in the experimental setup, the current passing through it may be increased indefinitely to achieve the desired magnetic field. Therefore, by increasing the current through the wire to 100 A, a magnetic field of 0.25 T is produced and the separation time would then be reduced to approximately 60 s. This calculation illustrates that while it is possible to exploit a spore's intrinsic magnetic properties for separation, it does require moderately strong magnetic fields (see Supplementary Figure S4 and Melnik et al., 2007). This analysis also disregards the fact that the calculated magnetic susceptibility is an average property and therefore natural variation will mean that longer times will be required to achieve high separation yields.

(iii) Adsorption: Manganese is accumulated both inside the spore and on its surface. while the presence of manganese in the core is well known (Thomas, 1964), adsorption to the spore integuments is less well characterized. Alderton and Snell (1963) demonstrated that bacterial spores behave like ion exchange gels, where following an acid regeneration treatment, the surface becomes free to take up structures that show base exchange behavior. The results in Figure 3a may be interpreted further using an adsorption model. Subtracting the manganese content of acid‐treated spores in Figure 3a from that of untreated spores yields an adsorption curve. Note that the concentration of manganese has been adjusted by approximately 43 µM in order to account for the onset of adsorption. By assuming that manganese adsorbs onto spores following the Langmuir isotherm, an inverse plot may be used to obtain the adsorption parameters *q*_m_ = 14.4 mg/g and *K*_D_ = 0.88 mM^−1^ (Supplementary Figure S3). Based on Figure 3a, the adsorption capacity was higher than expected, however, in the absence of competing ions it does appear to be a reasonable value. Compared with alternative natural manganese adsorbents (Omri & Benzina, 2012), a maximum adsorption capacity of 14.4 mg Mn^2+^ per gram of spore may not be particularly attractive. Equally, spores are not limited to the adsorption of manganese ions. The activated surface of acid treated spores, for example, may find an application in removing heavy metal ions from contaminated waters. Additionally, this work has shown that spores can be easily functionalized with ferromagnetic nanoparticles, greatly increasing their magnetic susceptibility and enabling their use in a wide array of biotechnological applications for example, drug delivery.

## 5 CONCLUSION

*Bacillus* spores are paramagnetic due to the high manganese content accumulated within the spore core and that associated with the spore coat (which may be surface bound or distributed throughout the coat). This manganese exists in different chemical states amongst spores of different species and the amount can be increased by approximately 10‐fold by altering the concentration of manganese in the culture media. The impact of these results on three spore‐related biotechnological applications have been considered with the most promising applications being associated with magnetic separations and metal ion adsorption. The spore's intrinsic magnetic strength was sufficient to allow for separation using moderately strong magnetic fields, while the possibility of adsorbing various metals onto its surface could enhance separation or enable spores in a variety of related applications such as drug delivery or bioremediation.

## CONFLICTS OF INTEREST

The authors have no conflicts of interest to declare.

## Supporting information

Additional Supporting Information may be found online in the supporting information tab for this article.

Supporting Data S1.
