# Supplementary material for: Paramagnetism in Bacillus spores: Opportunities for novel biotechnological applications
Source: Biotechnol Bioeng. 2017 Dec 15;115(4):955–64. doi: 10.1002/bit.26501 (PMC5888203; doi:10.1002/bit.26501)
Supplement: Supplementary file 1 — Supporting Data S1. [file BIT-115-955-s001.docx]

# Supplementary information

Table S1 Best fit parameters for field and temperature sweep measurements showing differences in manganese content (*N*) and magnetic moment (*m*) in *B. megaterium* spores grown at various temperatures, pH and manganese concentrations. Field sweep data have been fitted to Equation (1). Temperature sweep data have been fitted to Equation (3) and the corresponding *m* was calculated using Equation (4).

| **Variable** | **Value** | **Field sweep (*T* = 5 K)** | |  | **Temperature sweep (*H* = 0.1 T)** | |
| --- | --- | --- | --- | --- | --- | --- |
|  |  | ***N* (×10^22^ kg^-1^)** | ***m* (*μ*_B_)** |  | ***C* (m^3^ K kg^-1^)** | ***m* (*μ*_B_)** |
| **Standard** | 20 μM MnCl_2_, 30°C, pH 7.2 | 1.55 ± 0.08 | 5.9 ± 0.2 |  | 1.06 ± 0.03 | 5.7 ± 0.3 |
| **[MnCl_2_] (μM)** | 50 | 4.4 ± 0.1 | 5.7 ± 0.1 |  | 2.9 ± 0.1 | 5.6 ± 0.1 |
|  | 100 | 7.5 ± 0.8 | 5.6 ± 0.4 |  | 4.8 ± 0.1 | 5.5 ± 0.4 |
|  | 200 | 11.8 ± 0.4 | 5.5 ± 0.2 |  | 7.2 ± 0.2 | 5.4 ± 0.3 |
|  | 500 | 17.3 ± 0.6 | 5.3 ± 0.1 |  | 9.3 ± 0.3 | 5.1 ± 0.4 |
|  | 700 | 17.9 ± 1.7 | 5.1 ± 0.2 |  | 9.7 ± 0.2 | 5.1 ± 0.5 |
|  | 1000 | 18.1 ± 0.4 | 5.2 ± 0.1 |  | 9.8 ± 0.2 | 5.1 ± 0.2 |

Table S2 Best-fit parameters for field sweep measurements showing the manganese content (*N*) and magnetic moment (*m*) in *B. cereus* and *B. subtilis* spores. Field sweep data have been fitted to Equation (1) and the manganese content in spores was estimated according to Equation (2).

| **Species** | **Variable** | **Value** | **Field sweep (*T* = 5 K)** | |  | **Spore Mn content (wt%)** |
| --- | --- | --- | --- | --- | --- | --- |
|  |  |  | ***N* (×10^22^ kg^-1^)** | ***m*** |  |  |
| ***B. cereus*** | | | | | | |
|  | Standard | 10 μM MnCl_2_, 30°C, pH 7.2 | 2.3 ± 0.1 | 5.1 ± 0.2 |  | 0.21 ± 0.01 |
|  | [MnCl_2_]  (μM) | 100 | 4.93 ± 0.03 | 4.94 ± 0.02 |  | 0.45 ± 0.01 |
|  |  | 400 | 10.4 ± 0.1 | 4.51 ± 0.02 |  | 0.95 ± 0.01 |
|  |  | 600 | 17.5 ± 0.1 | 4.39 ± 0.04 |  | 1.60 ± 0.01 |
|  |  | 1000 | 21 ± 3 | 3.83 ± 0.3 |  | 1.9 ± 0.2 |
| ***B. subtilis*** | | | | | | |
|  | Standard | 50 μM MnCl_2_, 37°C, pH 7.2 | 4.2 ± 0.3 | 5.5 ± 0.3 |  | 0.38 ± 0.03 |

Table S3 Comparison of the quantity and magnetic moment of manganese in acid treated (labelled with an ‘A’) and untreated *B. megaterium* spores determined by field sweep measurements. Manganese content for a selection of spore samples was also measured commercially by ICP-OES.

| **[MnCl_2_] in culture media (μM)** | **Field sweep**  **(*T* = 5 K)** | | **Mn content measured by ICP-OES (wt. %)** |
| --- | --- | --- | --- |
|  | ***w*_Mn_ (wt. %)** | ***m* (*μ*_B_)** |  |
| 20 (standard) | 0.14 ± 0.01 | 5.9 ± 0.2 | 0.15 |
| 20A | 0.14 ± 0.01 | 5.8 ± 0.3 | 0.15 |
| 100 | 0.68 ± 0.07 | 5.6 ± 0.4 | 0.72 |
| 100A | 0.61 ± 0.04 | 5.4 ± 0.2 | – |
| 200 | 1.07 ± 0.03 | 5.5 ± 0.2 | 1.13 |
| 200A | 0.92 ± 0.05 | 5.4 ± 0.2 | 0.98 |
| 500 | 1.57 ± 0.06 | 5.1 ± 0.4 | – |
| 500A | 1.07 ± 0.12 | 5.1 ± 0.1 | – |
| 700 | 1.63 ± 0.16 | 5.1 ± 0.6 | – |
| 700A | 0.98 ± 0.07 | 5.4 ± 0.4 | – |
| 1000 | 1.65 ± 0.04 | 5.2 ± 0.1 | 1.53 |
| 1000A | 1.03 ± 0.07 | 5.3 ± 0.1 | 0.98 |


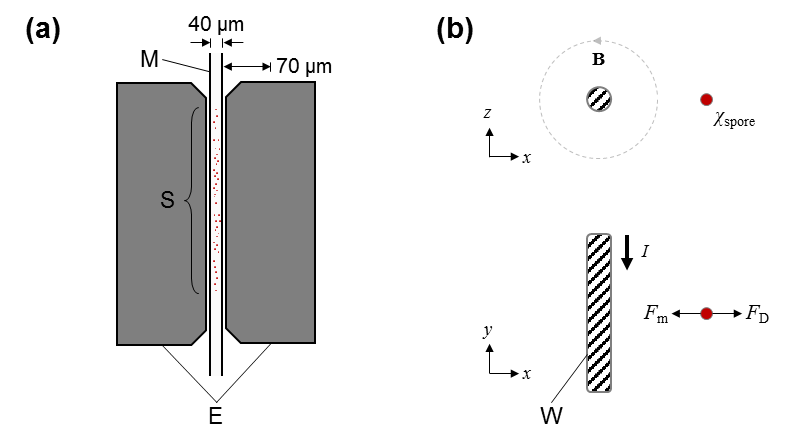


Figure S1 Set up and model of the microfluidic magnetic separation device. (a) schematic of the device used by [Siegel et al. (2006)](#_ENREF_2); (b) top and cut-through view of an idealised model for the device in (a) where the electromagnet has been replaced by a single wire carrying a current *I* which generates a magnetic field B equivalent to that generated by the electromagnet. *F*_m_ and *F*_D_ correspond to the magnetic and drag force, respectively. Figure adapted from [Shevkoplyas et al. (2007)](#_ENREF_1). Key: E – electromagnets; M – microfluidic channel; S – spores in deionised water; W – wire.


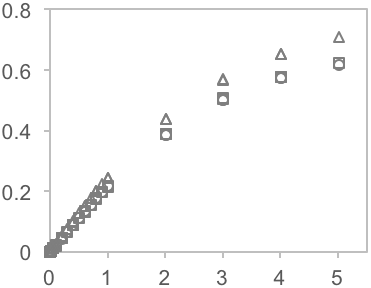

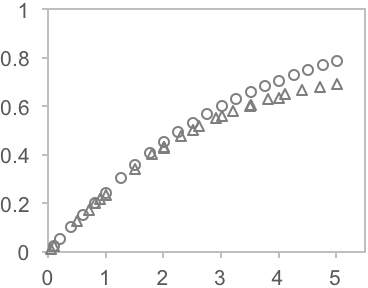


**(a)**

**(b)**

**Applied field** *μ*_0_**H** **(T)**

**M**

**(A m^2^ kg^-1^)**

**M**

**(A m^2^ kg^-1^)**

**Applied field** *μ*_0_**H** **(T)**

Figure S2 Plots comparing the differences in magnetisation for spores grown in culture media supplemented with divalent cations other than manganese. (a) *B. megaterium* spores: ○, standard conditions; △, 150 μM Fe^2+^; □, 150 μM Co^2+^; (b) *B. cereus* spores: ○, standard conditions; △, 200 μM Fe^2+^.


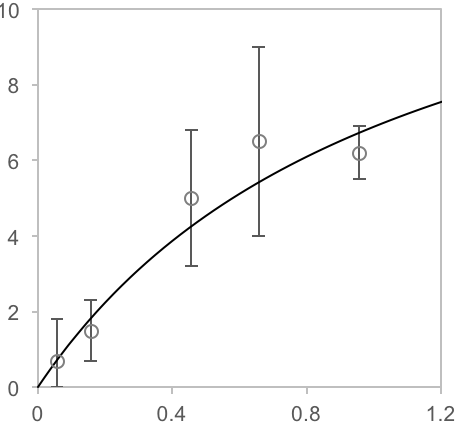

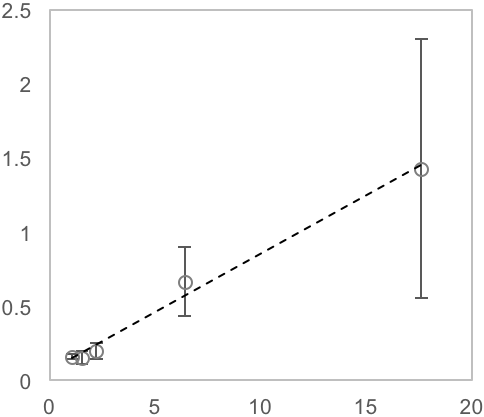


**(a)**

**(b)**

**Amount adsorbed, *q* (mg / g)**

***C*_Mn_ (mM)**

**1/*q* (g / mg)**

**1/*C*_Mn_ (mM^-1^)**

Figure S3 Data from Figure 3(a) replotted to show manganese adsorption characteristics for *B. megaterium* spores. (a) adsorption data fitted to a Langmuir isotherm (solid line) with parameters *q*_m_ = 14.4 mg/g and *K*_D_ = 0.88 mM^-1^; (b) reciprocal plot of (a) fitted to a line of best fit with equation y = 0.0788x + 0.0696.


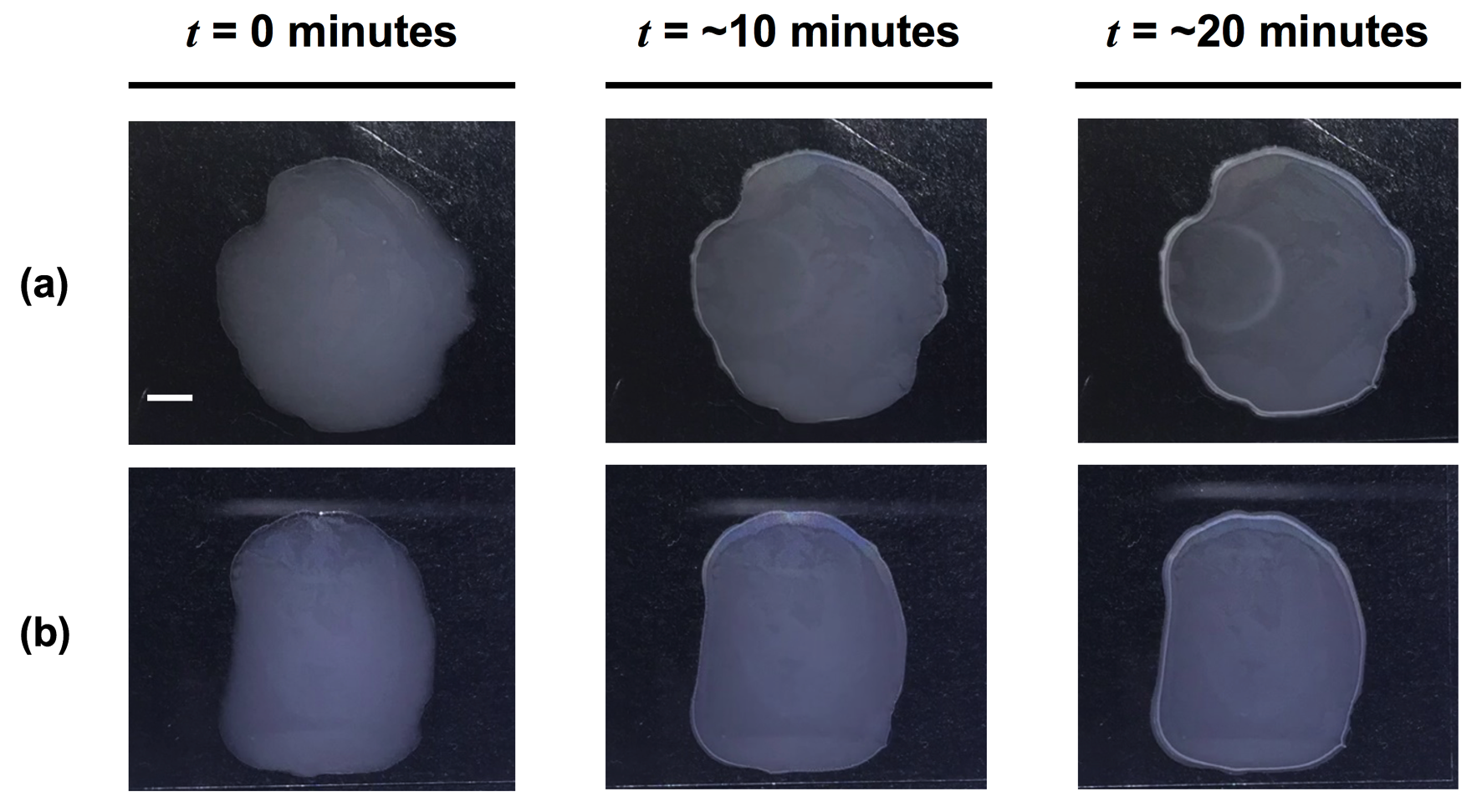


**Figure S4** Images demonstrating the effect of a permanent N42 grade neodymium magnet (remanence of ~1.3 T) on a 0.5 wt% suspension of *B. cereus* spores deposited on a glass coverslip; note that black tape was placed underneath the cover slip to increase contrast. (a) – a 5 mm diameter neodymium magnet was placed underneath the cover slip; (b) – no magnet was placed underneath the cover slip. The white line represents 2.5 mm and the scale is identical for all images.

Supplemental References

Shevkoplyas SS, Siegel AC, Westervelt RM, Prentiss MG, Whitesides GM. 2007. The force acting on a superparamagnetic bead due to an applied magnetic field. Lab on a Chip 7(10):1294-1302.

Siegel AC, Shevkoplyas SS, Weibel DB, Bruzewicz DA, Martinez AW, Whitesides GM. 2006. Cofabrication of electromagnets and microfluidic systems in poly(dimethylsiloxane). Angewandte Chemie International Edition 45(41):6877-6882.
